# Predictors of home-based cardiac rehabilitation exercise adherence among patients with chronic heart failure: a theory-driven cross-sectional study

**DOI:** 10.1186/s12912-023-01566-5

**Published:** 2023-11-06

**Authors:** Zhen Yang, Honghong Jia, Aiping Wang

**Affiliations:** 1 The First Affiliated Hospital of China Medical University, Shenyang, Liaoning China; 2 Department of Nursing, Harbin Medical University, Harbin, Heilongjiang China

**Keywords:** Cardiac rehabilitation, Social support, Fear of movement, Exercise self-efficacy, Adherence, Theory of planned behavior

## Abstract

**Background:**

The factors influencing home-based cardiac rehabilitation exercise adherence among patients with chronic heart failure remain unclear. This study aimed to explore predictors of home-based cardiac rehabilitation exercise adherence in these patients, based on the theory of planned behavior.

**Methods:**

This theory-driven, cross-sectional study used convenience sampling to recruit patients with chronic heart failure undergoing home-based cardiac rehabilitation. Instruments used included the Home-Based Cardiac Rehabilitation Exercise Adherence Scale, the Multidimensional Self-Efficacy for Exercise Scale, the Perceived Social Support Scale, and the Tampa Scale for Kinesiophobia Heart. Multivariate linear hierarchical regression analysis was employed to examine the factors influencing exercise adherence.

**Results:**

A total of 215 patients with chronic heart failure undergoing home-based cardiac rehabilitation participated in the study. The overall score for home cardiac rehabilitation exercise adherence was (48.73 ± 3.92). Multivariate linear hierarchical regression analysis revealed that age (*β*=-0.087, *p* = 0.012), education level (*β* = 0.080, *p* = 0.020), fear of movement (*β*=-0.254, *p* < 0.001), perceived social support (*β* = 0.451, *p* < 0.001), and exercise self-efficacy (*β* = 0.289, *p* < 0.001) influenced home-based cardiac rehabilitation exercise adherence. In the second model, fear of exercise explained 23.60% of the total variance, while perceived social support and exercise self-efficacy explained 26.60% of the total variance in the third model.

**Conclusion:**

This study found that home-based cardiac rehabilitation exercise adherence in patients with chronic heart failure was suboptimal, and identified its influencing factors. Targeted interventions addressing these factors, such as tailored education, support, and addressing fear of exercise, may help improve exercise adherence.

## Introduction

Chronic heart failure is a persistent clinical syndrome caused by structural and/or functional abnormalities of the heart and confirmed by objective evidence [1]. Despite some progress in prevention and control, the clinical prognostic burden of chronic heart failure, such as disability and adverse cardiovascular events, remains significant [2, 3]. Therefore, it is necessary to explore effective interventions to control chronic symptoms and improve clinical prognosis of patients with chronic heart failure.

Cardiac rehabilitation encompasses a variety of approaches designed to help individuals with heart disease achieve optimal physical, mental, and social functioning through their own efforts, thereby enabling them to lead active lives [4, 5]. Exercise-based cardiac rehabilitation has been confirmed to significantly enhance cardiopulmonary function and clinical prognosis in patients with chronic heart failure [6–8]. Nevertheless, due to the disease’s chronic nature, long-term outpatient rehabilitation exercises can incur substantial financial and time costs. As a meaningful alternative model [9, 10], unsupervised home-based cardiac rehabilitation has been found to yield comparable benefits to institutional cardiac rehabilitation in terms of improving clinical outcomes, mitigating cardiovascular risks, and cost-effectiveness. Moreover, a series of studies have corroborated that home-based cardiac rehabilitation can bolster exercise endurance and quality of life, foster mental well-being, and curtail readmission rates among patients with chronic heart failure [11–14].

However, reaping these benefits hinges on long-term adherence to home-based cardiac rehabilitation exercises. Regrettably, it has been reported that long-term adherence among patients with chronic heart failure remains suboptimal [15]. After completing institutional cardiac rehabilitation, only 52% of patients with chronic heart failure participate in home-based exercises [16]. Likewise, 43–63% of these patients are disinclined to engage in regular cardiac rehabilitation exercises due to the persistent nature of their condition and limited exercise endurance [17, 18]. Even with stringent interventions, adherence to long-term (24 months) home cardiac rehabilitation exercises is relatively low (34%) [19]. Similarly, patients demonstrate low adherence to unsupervised home-based cardiac rehabilitation exercise programs in China. According to a study published in the Chinese Journal of Cardiovascular Medicine [20], less than 50% of patients with chronic heart failure comply with the recommended frequency of cardiac rehabilitation exercises, which is three times weekly. Another study that followed up with 283 patients a month after their discharge found that only 64.66% demonstrated good compliance with unsupervised home-based cardiac rehabilitation exercises [21]. Chinese patients often perceive cardiac rehabilitation exercises as an“optional”supplementary therapy rather than an integral part of their treatment, especially in an unsupervised context [22]. Moreover, a lack of professional guidance and support may hinder patients’ confidence and skill development in adhering to the exercise program [23]. Currently, there is no effective solution addressing the lack of sustainability in home-based cardiac rehabilitation exercises, mainly due to unclear influencing factors. Consequently, it is essential to examine the critical determinants affecting patients’ adherence to home-based cardiac rehabilitation exercises, enabling targeted and precise interventions for those with chronic heart failure.

**Fig. 1:**
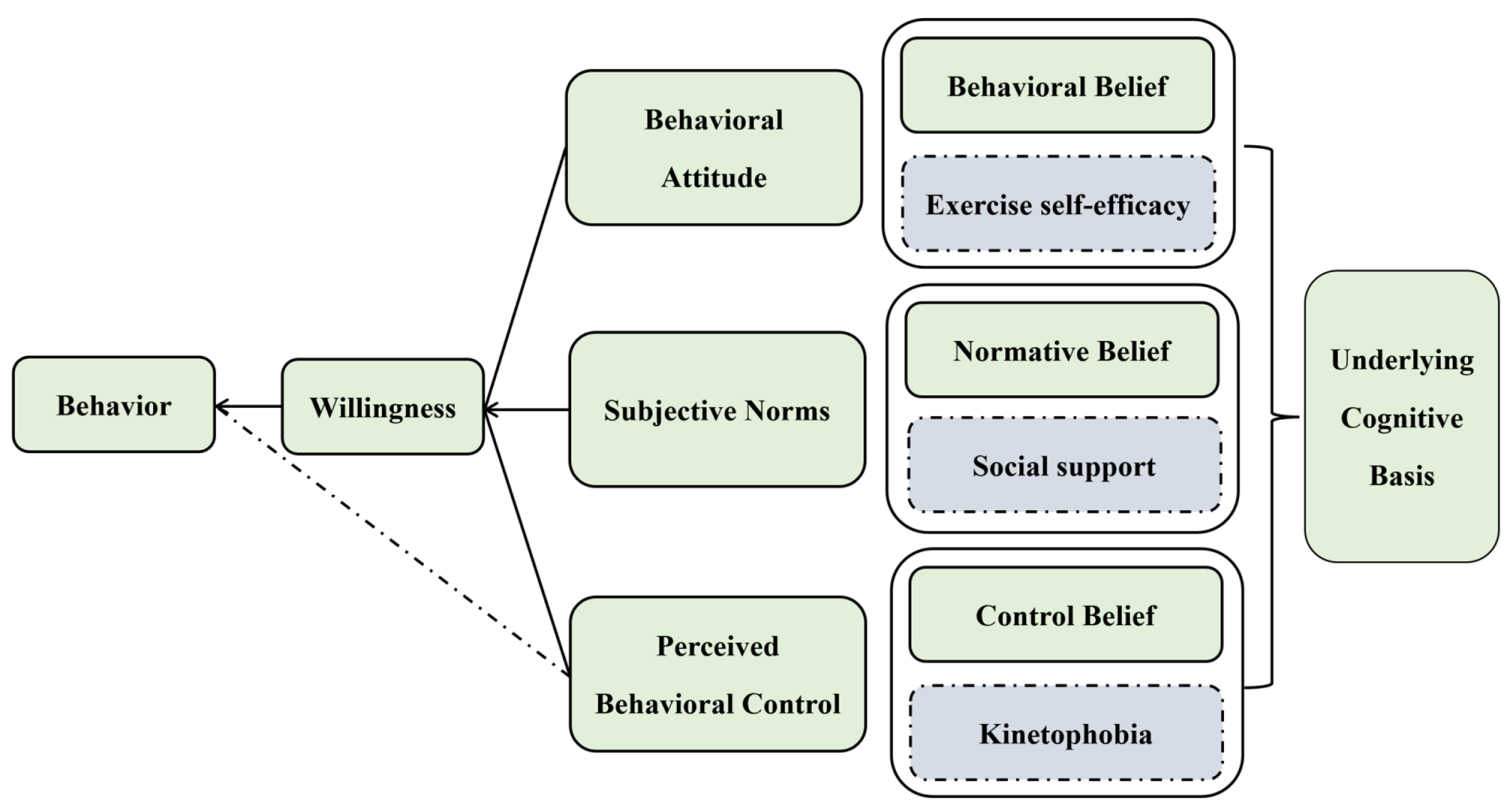
The theory of planned behavior framework with expected predictive variables

The theory of planned behavior, a behavioral theory, is useful for explaining and predicting home-based cardiac rehabilitation exercise adherence in patients with chronic heart failure. The theory of planned behavior emphasizes that individuals consider their behavioral intention before implementing a specific behavior, which is influenced by their behavioral attitude, subjective norms, and perceived behavioral control, all of which are affected by corresponding individual underlying cognitive basis (Fig. 1) [24]. Behavioral belief refers to an individual’s conscious tendency and confidence in achieving the desired outcomes (exercise self-efficacy in our study), while normative belief is defined as the external expectation for individuals to perform a specific behavior (perceived social support in our study) [24]. Control belief, on the other hand, is regarded as factors that individuals perceive to facilitate or hinder their behavior (exercise fear in our study) [24]. These three beliefs form the foundation and core of the theory of planned behavior, providing a suitable theoretical basis for exploring the predictors of home-based cardiac rehabilitation exercise adherence in patients with chronic heart failure (Fig. 1).

Consequently, this study, as a component of a broader research project, aimed to explore the predictors for home-based cardiac rehabilitation exercise adherence among patients with chronic heart failure. The study focused on the three potential core beliefs (behavioral beliefs, normative beliefs, and control beliefs) derived from the theory of planned behavior, with the intent of identifying targets for intervention and providing a reference for the development of accurate and targeted interventions.

## Methods

### Participants and samples

This cross-sectional observational study recruited eligible patients with chronic heart failure from a large cardiac rehabilitation center in Liaoning Province, mainland China. The patients were selected using convenient sampling methods during their outpatient clinic visits. The inclusion criteria for participants were: (a) 18 years of age and above; (b) undergoing unsupervised home-based cardiac rehabilitation (they were performing an unsupervised home-based cardiac rehabilitation exercise prescribed by a cardiac rehabilitation physician); (c) voluntary participation in the study. The exclusion criteria were: (a) patients with mental illnesses rendering them unable to communicate normally; (b) patients with other severe organic diseases (e.g. patients with chronic heart failure requiring neurological and pulmonary rehabilitation such as stroke and chronic obstructive pulmonary disease). The sample size was determined using the G-power 3.1.9.7 procedure. To identify predictors of home-based cardiac rehabilitation exercise adherence, the following parameters were set: a medium effect size (f ^2^) of 0.15, a significance level (α) of 0.05, statistical power (1 − β) of 0.90, and 15 predictors (seven predictive variables from the Socio-Demographic Characteristics Questionnaire, four dimensional variables from the Tampa Scale for Kinesiophobia Heart, and three dimensional variables each from the Perceived Social Support Scale and the Multidimensional Self-Efficacy for Exercise Scale). The minimum required sample size was calculated as 171 cases. To account for a 20% sampling error, the minimum target sample size was set at 206.

### Instruments

#### Socio-demographic characteristics Questionnaire

After a literature review and group discussion, the questionnaire was developed to collect the general socio-demographic characteristics of the participants. The questionnaire covers gender, age, marital status, educational level, duration of disease, monthly income, and place of residence.

#### Home-based Cardiac Rehabilitation Exercise Adherence Scale

Building on prior constructivist grounded theory research [25], we previously developed a scale to measure home-based cardiac rehabilitation exercise adherence among patients with cardiovascular disease [26]. The scale encompasses 20 items and 4 dimensions, including seeking supports, rehabilitation exercise, exercise monitoring, and information feedback. We utilized a Likert 5-point rating system to collect participants’ responses. The scale ranges from 20 to 100 points, with higher scores indicating better adherence to home-based cardiac rehabilitation exercises. A score of 60 or less is considered low adherence, and a score greater than 60 is considered high adherence.

#### Tampa Scale for Kinesiophobia Heart

The original scale was developed to assess exercise fear levels in patients with coronary heart disease [27]. Subsequently, this scale has been widely used in patients with chronic heart failure and proved to have good psychometric properties [28, 29]. In this study, the Chinese version of the Tampa Scale for Kinesiophobia Heart was adopted [30]. The scale includes 17 items and 4 dimensions covering perceived danger for heart problem (danger), fear of injury (fear), avoidance of exercise (avoidance), and dysfunctional self (dysfunction). A Likert 4-point rating system was adopted to collect participants’ responses. The scale ranges from 17 to 68 points, with higher scores representing higher levels of exercise fear. In this study, the Cronbach’α coefficient of the scale was 0.790.

#### Perceived Social Support Scale

In this study, perceived social support in patients with chronic heart failure was measured by the Perceived Social Support Scale [31]. The Chinese version of Perceived Social Support Scale has appropriate reliability and validity [32]. The scale is a 12-item self-report tool covering family support, friend support, and other support. A 7-point Likert rating system was adopted to receive responses from participants. The scale has an overall score ranging from 12 to 84, with higher scores representing higher levels of perceived support. In this study, the Cronbach’α coefficient of the scale was 0.774.

#### Multidimensional self-efficacy for Exercise Scale

The original scale was developed to measure patients’ confidence in exercise [33]. In this study, the Chinese version of the Multidimensional Self-Efficacy for Exercise Scale was adopted [34]. This scale includes 9 items and 3 dimensions, covering task efficacy, coping efficacy and scheduling efficacy. In addition, each item is rated on a scale of 0 to 10, with 0 being no confidence and 10 being completely confident. The higher the score, the more confident the patient was to keep exercising. In this study, the Cronbach’α coefficient of the scale was 0.763.

### Data collection

After being fully informed of the purpose and significance of the study, as well as its voluntary and anonymous nature, 220 patients living with chronic heart failure were invited to participate in the research program during their outpatient follow-ups, with the assistance of clinical nurses. A remarkable 97.73% of them (215 patients) agreed to take part and filled out the questionnaires anonymously in a quiet space after signing an informed consent form. For those with poor eyesight or those who could not fill out the questionnaire on their own, the researchers carefully read out each item one by one. The patients then answered each question verbally, and the researchers recorded their responses. All completed questionnaires were checked thoroughly on site to ensure that the results were accurate and authentic.

### Data analysis

In this study, data analysis was performed using SPSS 26.0 software. Continuous variables were presented as mean and standard deviation, while categorical variables were expressed in terms of frequency and proportion. To compare the differences in home-based cardiac rehabilitation exercise adherence across various social demographics, the t-test and analysis of variance were employed. A multiple linear hierarchical stepwise regression analysis was conducted to investigate the factors affecting home-based cardiac rehabilitation exercise adherence in patients with chronic heart failure, based on the theory of planned behavior. A p-value of less than 0.05 was considered statistically significant.

### Ethical consideration

All procedures were conducted in accordance to the Declaration of Helsinki of 1964 and its further modifications. Participants were informed of the purpose of the study, the confidentiality of the data, and the voluntary nature of their participation. All participants gave informed forms. The research proposal was approved by the Ethics Review Committee of the First Affiliated Hospital of China Medical University (No. 2023. 66).

## Results

### Socio-demographic characteristics

The socio-demographic characteristics of the participants are displayed in Table 1. In this study, there were 103 men and 112 women with a mean age of 60.78 years and a standard deviation (SD) of 2.34. A majority of the participants were married (53.5%) and had a monthly income of less than 3,000 RMB (52.1%). Less than half of the participants had completed a secondary education (42.3%) and had been living with the disease for less than 4 years (41.9%).

**Table 1:** Intergroup differences in based-home cardiac rehabilitation exercise adherence

Variable	Frequency (%)	Mean (standard deviation)	Statistical value	p-value
Age			23.867	<0.001
<60	55 (25.6)	51.29 (4.03)		
60 ~ 70	85 (39.5)	48.66 (2.78)		
>70	75 (34.9)	46.93 (3.95)		
Gender			-1.089	0.278
Male	103 (47.9)	48.43 (4.07)		
Female	112 (52.1)	49.01 (3.77)		
Educational level			24.732	<0.001
Primary education	86 (40.0)	47.28 (3.69)		
Secondary education	91 (42.3)	48.68 (3.12)		
Higher education	38 (17.7)	52.13 (4.11)		
Marital status			2.785	0.064
Unmarried	20	47.30 (5.97)		
Married	115	48.50 (3.09)		
Divorced/Widowed	80	49.41 (4.26)		
Duration of disease (year)			0.479	0.620
<4	90 (41.9)	48.97 (4.02)		
4 ~ 8	87 (40.5)	48.41 (3.98)		
>8	38 (17.7)	48.89 (3.57)		
Monthly income (RMB)			1.506	0.224
<3000	112 (52.1)	48.46 (3.81)		
3000 ~ 6000	60 (27.9)	48.57 (4.33)		
>6000	43 (20.0)	49.65 (3.53)		
Place of residence			-2.545	0.012
Rural	75 (34.9)	47.81 (3.63)		
Urban	140 (65.1)	49.22 (3.99)		
Number of chronic diseases			1.068	0.345
<3	80 (37.2)	48.24 (3.82)		
3~4	102 (47.4)	49.09 (3.96)		
>4	33 (15.3)	48.82 (4.00)		

### Home-based cardiac rehabilitation exercise adherence

The overall score for home cardiac rehabilitation exercise adherence in patients with chronic heart failure was (48.73 ± 3.92). The mean scores for the four dimensions covering seeking supports, rehabilitation exercise, exercise monitoring, and information feedback were (12.39 ± 1.78), (12.38 ± 2.00), (11.89 ± 1.70), and (12.07 ± 2.16), respectively.

### Socio-demographic differences in based-home cardiac rehabilitation exercise adherence

The between-group differences in home-based cardiac rehabilitation exercise adherence scores are presented in Table 1. The results revealed that the differences in home-based cardiac rehabilitation exercise adherence scores among ages, education levels, and place of residence were statistically significant (*p* < 0.05).

### The correlations between variables

The correlations between various variables in this study are shown in Table 2. The results demonstrated that the total score of exercise self-efficacy (*r* = 0.377 ~ 0.783, *p* < 0.001) and the total score of perceived social support (*r* = 0.272 ~ 0.688, *p* < 0.001) were positively correlated with the total score of home-based cardiac rehabilitation exercise adherence and its dimensional scores. The total score of fear of movement (*r* = 0.259 ~ 0.663, *p* < 0.001) was negatively correlated with the total score of home-based cardiac rehabilitation exercise adherence and its dimensional scores.

**Table 2:** The correlations between various variables in this study

Variable	Exercise adherence	Seeking supports	Rehabilitation exercise	Exercise monitoring	Information feedback
Exercise self-efficacy	0.783^**^	0.379^**^	0.428^**^	0.377^**^	0.414^**^
Task efficacy	0.612^**^	0.279^**^	0.421^**^	0.287^**^	0.262^**^
Scheduling efficacy	0.634^**^	0.318^**^	0.333^**^	0.311^**^	0.334^**^
Coping efficacy	0.677^**^	0.333^**^	0.297^**^	0.329^**^	0.419^**^
Perceived social support	0.688^**^	0.360^**^	0.414^**^	0.272^**^	0.353^**^
Family support	0.490^**^	0.223^**^	0.293^**^	0.171^**^	0.298^**^
Friends support	0.537^**^	0.303^**^	0.323^**^	0.207^**^	0.262^**^
Significant other	0.570^**^	0.309^**^	0.345^**^	0.253^**^	0.259^**^
Exercise fear	-0.682^**^	-0.366^**^	-0.381^**^	-0.348^**^	-0.307^**^
Danger	-0.537^**^	-0.305^**^	-0.314^**^	-0.291^**^	-0.201^**^
Fear	-0.507^**^	-0.270^**^	-0.283^**^	-0.217^**^	-0.263^**^
Avoidance	-0.628^**^	-0.349^**^	-0.360^**^	-0.309^**^	-0.274^**^
Dysfunction	-0.553^**^	-0.269^**^	-0.282^**^	-0.322^**^	-0.265^**^

Note. ^**^ indicates significance p < 0.001. Row covers the dependent variables (exercise adherence) and its dimensions, and column covers the independent variables (exercise self-efficacy, perceived social support, and exercise fear) and its dimensions

### Predictors of home-based cardiac rehabilitation exercise adherence

Based on theoretical considerations [24] and literature review, we select three blocks for the stepwise order for entering of predictors. Block 1 contained the general demographic information of the participants. Considering that exercise fear was identified as the strongest predictor of exercise adherence in previous studies, block 2 contained only exercise fear (R^2^ increased beyond general demographic variables). In addition, block 3 contained perceived social support and exercise self-efficacy (R^2^ increase after accounting for the influence of exercise fear). Based on the results of univariate analysis and correlation analysis, 6 of 15 variables were selected for hierarchical stepwise regression analysis. In this study, the significant hierarchical stepwise regression analysis model includes 5 of 6 eligible variables, covering age (*β*=-0.087, *p* = 0.012), education level (*β* = 0.080, *p* = 0.020), fear of movement (*β*=-0.254, *p* < 0.001), perceived social support (*β* = 0.451, *p* < 0.001), and exercise self-efficacy (*β* = 0.289, *p* < 0.001) (Table 3). In addition, the explained variance was 79.3% in the final block 3. Exercise fear explained 23.6% of the total variance, while perceived social support and exercise self-efficacy explained 26.6% of the total variance (Table 3).

**Table 3:** The results of the multiple linear hierarchical stepwise regression analysis

Variables	Block 1^a^		Block 2^b^		Block 3^c^	
	*β* (95% *CI*)	*p*-value	*β* (95% *CI*)	*p*-value	*β* (95% *CI*)	*p*-value
Age	-0.339 (-0.457, -0.222)	<0.001	-0.196 (-0.296, -0.197)	<0.001	-0.087 (-0.154, -0.019)	0.012
Education level	0.323 (0.205, 0.440)	<0.001	0.177 (0.077, 0.276)	<0.001	0.080 (0.013, 0.147)	0.020
Place of residence	0.127 (0.013, 0.241)	0.029	0.035 (-0.060, 0.129)	0.473	0.018 (-0.045, 0.080)	0.578
Exercise fear			-0.548 (-0.652, -0.443)	<0.001	-0.254 (-0.332, -0.177)	<0.001
Exercise self-efficacy					0.289 (0.213, 0.364)	<0.001
Perceived social support					0.451 (0.373, 0.529)	<0.001
R^2^	0.301		0.536		0.798	
Adjusted R^2^	0.291		0.527		0.793	
F value	<0.001		<0.001		<0.001	

Note. ^a^ Predictors: age, education level, place of residence (block 1). ^b^ Predictors: age, education level, place of residence (block 1), exercise fear (block 2). ^c^ Predictors: age, education level, place of residence (block 1), exercise fear (block 2), exercise self-efficacy, perceived social support (block 3)

## Discussion

The practice of home-based cardiac rehabilitation exercise is an essential component in the healthcare management for patients experiencing chronic heart failure [35]. Given that sustained adherence is fundamental to realizing the numerous potential benefits of such practices [36], this study delves deeply into the factors that influence adherence to home-based cardiac rehabilitation among these patients. Strong and growing evidence supports the efficacy of home-based cardiac rehabilitation, citing not only the potential to improve the physiological condition of patients but also the capacity to enhance the quality of life, psychological wellbeing, and health behaviors [11, 37, 38]. The study recognizes the importance of customization, portability, and individual patient autonomy offered by home-based cardiac rehabilitation. It capitalizes on these benefits without sacrificing the clinical supervision that conventional center-based cardiac rehabilitation provides [39]. Identifying targeted factors, this study addresses the interplay of both individual internal resources (like the psychological readiness to adhere) and external resources (like the availability of a conducive home environment or adequate supervision). This comprehensive assessment paves the way for the development of systematic and precise interventions. Through careful adjustments and modifications in an individual’s recovery journey, the long-term adherence to and benefits from home-based cardiac exercise are significantly enhanced.

The findings of this study showed that the level of home-based cardiac rehabilitation exercise adherence among patients with chronic heart failure is relatively low. In terms of various dimensions, the levels of seeking supports, rehabilitating exercise, exercise monitoring, and information feedback are also suboptimal. Seeking supports is the initial adherence behavior of patients with chronic heart failure in home-based cardiac rehabilitation exercise [25]. Disease-related stigma is considered an essential factor hindering patients from seeking supports [40]. Patients with chronic heart failure who experience this stigma may be reluctant to seek supports from others during home-based cardiac rehabilitation exercise, resulting in lower adherence to seeking support. Rehabilitating exercise is the core adherence behavior of patients with chronic heart failure in home-based cardiac rehabilitation exercise [25]. Due to the chronic nature of the disease, patients with chronic heart failure suffer from prolonged adverse symptoms. Consequently, their fear of exercise during home-based cardiac rehabilitation may significantly weaken their courage and confidence in completing these activities [41]. Exercise monitoring is the key adherence behavior of patients with chronic heart failure in home-based cardiac rehabilitation exercise [25]. Limited knowledge may be a significant factor in the low adherence to exercise monitoring among chronic heart failure patients [42]. Those with limited knowledge may not realize the importance of exercise monitoring or recognize the monitoring data. Information feedback is the driving adherence behavior of patients with chronic heart failure in home-based cardiac rehabilitation exercise [25]. Patients with chronic heart failure need to participate in outpatient follow-ups to provide feedback on their home-based cardiac rehabilitation exercises over time. However, a variety of objective barriers, such as limited financial resources and poor transportation, or subjective barriers as measured by the Cardiac Rehabilitation Barriers Scale, may significantly reduce their adherence to outpatient follow-ups for providing relevant feedback [43–45]. In summary, for patients with chronic heart failure exhibiting low adherence to home-based cardiac rehabilitation exercise, a series of brief intervention strategies (e.g., contact and education) can be employed, considering the above-mentioned modifiable factors, to enhance their adherence to home-based cardiac rehabilitation exercise.

The results of this study indicate that age and educational level are predictive factors affecting the adherence of chronic heart failure patients to home-based cardiac rehabilitation exercises, consistent with the findings of Pedersen et al. [46]. The older the chronic heart failure patient, the lower the adherence to home-based cardiac rehabilitation exercises. The coexistence of chronic diseases leads to limited exercise endurance in older chronic heart failure patients. Due to physical discomfort, these patients are unwilling to engage in cardiac rehabilitation exercises at home and prefer to rest instead [47]. On the other hand, chronic heart failure patients with a higher level of education exhibit increased adherence to home-based cardiac rehabilitation exercises. According to the knowledge-attitude-practice theory [48], knowledge serves as the foundation for generating attitude, and attitude act as the driving force for individuals to produce practice. Patients with a higher level of education may possess more knowledge about cardiac rehabilitation through internal and external resources [49]. The benefits of cardiac rehabilitation exercises will encourage them to develop positive beliefs, resulting in subsequent participation in these exercises. Based on these findings, during outpatient follow-up periods, particular attention should be given to older chronic heart failure patients and those with lower educational levels, with an emphasis on improving their adherence to home-based cardiac rehabilitation exercises through effective methods of emotional regulation and cognitive education.

In this study, fear of movement is hypothesized and proved to be a control belief within the framework of planned behavior theory. In line with previous research findings [50], fear of movement serves as an independent negative predictor of home-based cardiac rehabilitation exercise adherence among chronic heart failure patients, accounting for 23.6% of the total variation. The higher the level of fear of movement in chronic heart failure patients, the poorer their adherence to home-based cardiac rehabilitation exercises. Chronic heart failure patients experience long-term adverse clinical symptoms such as breathing difficulties and limited exercise endurance [51]. Patients with chronic heart failure may have high fear of exercise due to the aforementioned obstacles. Although these symptoms may be effectively controlled and monitored remotely by physicians, the long-term fear of exercise among patients with chronic heart failure will not be easily eliminated and may prevent them from engaging in beneficial exercise. Among them, exercise self-efficacy may play a strong mediating role [52]. Patients with chronic heart failure with high fear of exercise have a certain negative confidence in completing exercise prescriptions and may not be able to comply with home-based cardiac rehabilitation exercises [53]. Therefore, emotional regulation is indispensable for enhancing the adherence to home-based cardiac rehabilitation exercises among chronic heart failure patients who experience fear of movement.

In addition, the present study’s findings reveal that exercise self-efficacy and perceived social support are independent driving factors affecting the adherence of chronic heart failure patients to home-based cardiac rehabilitation exercises, together accounting for 26.60% of the total variance. Exercise self-efficacy refers to individuals’ belief in their ability and practice to perform physical activities [54]. In this study, exercise self-efficacy is considered as a behavioral belief within the framework of theory of planned behavior. The higher the exercise self-efficacy, the better the adherence of chronic heart failure patients to home-based cardiac rehabilitation exercises. According to the information-motivation-behavioral skills model [55], chronic heart failure patients with higher exercise self-efficacy may possess stronger exercise beliefs and motivation, be more willing to engage in home-based cardiac rehabilitation exercises, or more eager to develop rehabilitation exercise skills or overcome related obstacles [56]. Notably, this represents a positive reinforcement cycle for adherence to home-based cardiac rehabilitation exercises. Based on these findings, exercise self-efficacy may be regarded as a potential target for developing interventions aimed at improving the adherence of chronic heart failure patients to home-based cardiac rehabilitation exercises. Perceived social support refers to the assistance that individuals perceive they receive from external resources in a specific situation [57]. In this study, perceived social support is considered as a normative belief within the framework of the theory of planned behavior. The higher the perceived social support, the better the adherence of chronic heart failure patients to home-based cardiac rehabilitation exercises. Home-based cardiac rehabilitation exercises constitute a highly customized behavioral program, requiring patients to follow prescriptions provided by cardiac rehabilitation professionals [58]. In this process, supports from family members, peers, and professionals is essential for enhancing adherence to home-based cardiac rehabilitation exercises [59]. With the psychological support of family, the experiential support of peers, and the supervisory support from professionals, chronic heart failure patients can gain higher confidence and courage to complete the prescribed cardiac rehabilitation exercise tasks at home [60]. This remains a positive reinforcement cycle for exercise adherence. Therefore, social support may serve as a powerful external resource for improving adherence to home-based cardiac rehabilitation exercises among chronic heart failure patients. Different aspects of support from family, peers, and professionals should be incorporated into the home-based cardiac rehabilitation program to enhance patients’ adherence.

Our study is grounded on the central beliefs of the theory of planned behavior, covering behavioral, normative, and control beliefs. The theory of planned behavior serves as a comprehensive framework for forecasting health-related actions at an individual level. In this context of our research, we proposed that the degree of belief in one’s capability to exercise successfully (exercise self-efficacy), the level of support a person feels they receive from others (perceived social support) and fear of exercising (exercise fear), each correlating with behavioral, normative, and control beliefs respectively, could be the significant predictors of adherence to exercise regimen in patients experiencing chronic heart failure. Confirming our hypothesis, our primary results showed that exercise self-efficacy, perceived social support, and exercise fear - each grounded in the theory of planned behavior - emerge as forceful predictors, and collectively explain 50.20% of fluctuation in commitment to exercise amongst patients experiencing chronic heart failure. Specifically, the theory of planned behavior suggests that those who have faith in their power to carry out the exercise (a high degree of exercise self-efficacy) are more prone to stick to a home-based cardiac rehabilitation program. Additionally, our study found that individuals who felt a greater amount of social support were more often compliant with an exercise regime. On the other hand, fear of exercise could discourage patients from participating in an exercise regime, indicating a lower extent of perceived behavioral control and as a result, causing lesser adherence. Overall, these explanations not only stress the significance of our findings but also spotlight the critical role of the theory of planned behavior in understanding and predicting health-oriented behaviors among patients suffering from chronic heart failure.

### Limitation

There are several limitations that need to be discussed in this study. First, a larger, multi-center sample should be considered to supplement the results of this study, although the sample size is sufficient for the current investigation. Furthermore, biases arising from convenience sampling are unavoidable. As a result, a 20% sampling error is taken into account in this study. Lastly, the predictive factors for adherence to home-based cardiac rehabilitation exercise among chronic heart failure patients have been identified, but the causal relationship pathways have not yet been explored. This will be considered in our future research. In addition, limited predictors were included in this study, and a more comprehensive exploration of predictors of adherence to home-based cardiac rehabilitation exercise in patients with chronic heart failure is also the focus of our future research work.

## Conclusion

This study investigates the factors affecting home-based cardiac rehabilitation exercise adherence among patients with chronic heart failure. Adherence to home-based cardiac rehabilitation exercises in these patients is found to be at a relatively low level. Special attention should be given to older patients and those with lower educational attainment. Additionally, social support and self-efficacy may serve as potential intervention targets to improve the adherence of chronic heart failure patients to home-based cardiac rehabilitation exercises.

## Data Availability

The datasets used and/or analysed during the current study are available from the corresponding author on reasonable request.

## References

[1] Crea F (2023). Epidemiology and treatment of acute and chronic Heart Failure. Eur Heart J.

[2] Lorenzovici L, Bârzan-Székely A, Farkas-Ráduly S (2022). Burden of Chronic Heart Failure in Romania. Healthc (Basel).

[3] Butler J, Djatche LM, Sawhney B (2020). Clinical and economic Burden of Chronic Heart Failure and reduced ejection Fraction following a worsening Heart Failure event. Adv Ther.

[4] Josephson RA (2022). Cardiac rehabilitation 2022. Prog Cardiovasc Dis.

[5] Baman JR, Sekhon S, Maganti K, Cardiac Rehabilitation (2021). JAMA.

[6] Williamson T, Moran C, Chirico D (2021). Cancer and Cardiovascular Disease: the impact of cardiac rehabilitation and cardiorespiratory fitness on survival. Int J Cardiol.

[7] Lee JH, Kim J, Sun BJ, Jee SJ, Park JH (2021). Effect of Cardiac Rehabilitation on Left ventricular diastolic function in patients with Acute Myocardial Infarction. J Clin Med.

[8] Kamiya K, Sato Y, Takahashi T (2020). Multidisciplinary Cardiac Rehabilitation and Long-Term prognosis in patients with Heart Failure. Circ Heart Fail.

[9] Clark RA, Conway A, Poulsen V, Keech W, Tirimacco R, Tideman P (2015). Alternative models of cardiac rehabilitation: a systematic review. Eur J Prev Cardiol.

[10] Batalik L, Filakova K, Sladeckova M, Dosbaba F, Su J, Pepera G (2023). The cost-effectiveness of exercise-based cardiac telerehabilitation intervention: a systematic review. Eur J Phys Rehabil Med.

[11] Zwisler AD, Norton RJ, Dean SG (2016). Home-based cardiac rehabilitation for people with Heart Failure: a systematic review and meta-analysis. Int J Cardiol.

[12] Schopfer DW, Whooley MA, Allsup K (2020). Effects of Home-based Cardiac Rehabilitation on Time to Enrollment and functional status in patients with Ischemic Heart Disease. J Am Heart Assoc.

[13] Ramachandran HJ, Jiang Y, Tam WWS, Yeo TJ, Wang W (2022). Effectiveness of home-based cardiac telerehabilitation as an alternative to phase 2 cardiac rehabilitation of coronary Heart Disease: a systematic review and meta-analysis. Eur J Prev Cardiol.

[14] Krishnamurthi N, Schopfer DW, Shen H, Whooley MA (2019). Association of Mental Health Conditions with Participation in Cardiac Rehabilitation. J Am Heart Assoc.

[15] Ruano-Ravina A, Pena-Gil C, Abu-Assi E (2016). Participation and adherence to cardiac rehabilitation programs. A systematic review. Int J Cardiol.

[16] Schopfer DW, Nicosia FM, Ottoboni L, Whooley MA (2020). Patient perspectives on declining to Participate in Home-based Cardiac Rehabilitation: a MIXED-METHODS STUDY. J Cardiopulm Rehabil Prev.

[17] Kulnik ST, Sareban M, Höppchen I (2022). Outpatient cardiac rehabilitation closure and home-based exercise training during the first COVID-19 lockdown in Austria: a mixed-methods study. Front Psychol.

[18] Krishnamurthi N, Schopfer DW, Ahi T (2019). Predictors of patient participation and completion of home-based Cardiac Rehabilitation in the Veterans Health Administration for patients with Coronary Heart Disease. Am J Cardiol.

[19] Whellan DJ, O’Connor CM, Lee KL (2007). Heart Failure and a controlled trial investigating outcomes of exercise training (HF-ACTION): design and rationale. Am Heart J.

[20] Zhang Y, Wang Y, Li X, Tang X, Gu X, Yan H (2020). Factors associated with exercise adherence among patients with Heart Failure in China. Chin J Cardiovasc Med.

[21] Cao Q, Xu L, Wen S, Li F (2021). Investigating the influence of the Shared decision-making perception on the patient adherence of the home- and Exercise-based Cardiac Rehabilitation after Percutaneous Coronary intervention. Patient Prefer Adherence.

[22] Ma J, Ge C, Shi Y (2021). Chinese Home-based Cardiac Rehabilitation Model delivered by Smartphone Interaction improves clinical outcomes in patients with Coronary Heart Disease. Front Cardiovasc Med.

[23] Tadas S, Coyle D (2020). Barriers to and facilitators of technology in Cardiac Rehabilitation and Self-Management: systematic qualitative grounded theory review. J Med Internet Res.

[24] Bosnjak M, Ajzen I, Schmidt P (2020). The theory of Planned Behavior: selected recent advances and applications. Eur J Psychol.

[25] Yang Z, Sun L, Sun Y, Dong Y, Wang A (2023). A conceptual model of home-based Cardiac Rehabilitation Exercise Adherence in patients with chronic Heart Failure: a Constructivist grounded Theory Study. Patient Prefer Adherence.

[26] Yang Z, Sun Y, Wang H, Zhang C, Wang A (2023). A scale for measuring home-based cardiac rehabilitation exercise adherence: a development and validation study. BMC Nurs.

[27] Bäck M, Jansson B, Cider A, Herlitz J, Lundberg M (2012). Validation of a questionnaire to detect kinesiophobia (fear of movement) in patients with coronary artery Disease. J Rehabil Med.

[28] Qin J, Xiong J, Wang X, Gao Y, Gong K (2022). Kinesiophobia and its association with fatigue in CHF patients. Clin Nurs Res.

[29] Yu T, Gao M, Sun G, Graffigna G, Liu S, Wang J (2023). Cardiac rehabilitation engagement and associated factors among Heart Failure patients: a cross-sectional study. BMC Cardiovasc Disord.

[30] Lei MJ, Liu TT, Xiong SQ, Sang M, Jin CD (2019). Reliability and validity test of Chinese version of the Tampa Scale for Kinesiophobia Heart. Chin Nurs Manage.

[31] Zimet GD, Dahlem NW, Zimet SG, Farley GK. The multidimensional scale of perceived social support. J Pers Assess. 1988;52(1):30–41. 10.1207/s15327752jpa5201_2.

[32] Chou KL (2000). Assessing Chinese adolescents’ social support: the Multidimensional Scale of Perceived Social Support. Pers Individ Dif.

[33] Rodgers WM, Wilson PM, Hall CR, Fraser SN, Murray TC (2008). Evidence for a multidimensional self-efficacy for exercise scale. Res Q Exerc Sport.

[34] Dong J, Wang J, Yang H, Chang W, Liu H, Yu M, Ma Y (2022). Sinicization of the multidimensional self-efficacy for exercise scale and reliability and validity test in patients with coronary Heart Disease. Chin Nurs Res.

[35] Nagatomi Y, Ide T, Higuchi T (2022). Home-based cardiac rehabilitation using information and communication technology for Heart Failure patients with frailty. ESC Heart Fail.

[36] Kawada T (2022). Clinical benefits in patients with home-based cardiac rehabilitation in the era of COVID-19 pandemic. Heart Lung.

[37] Antoniou V, Davos CH, Kapreli E, Batalik L, Panagiotakos DB, Pepera G (2022). Effectiveness of home-based cardiac rehabilitation, using wearable sensors, as a multicomponent, cutting-edge intervention: a systematic review and meta-analysis. J Clin Med.

[38] Batalik L, Pepera G, Su JJ (2022). Cardiac telerehabilitation improves lipid profile in the long term: insights and implications. Int J Cardiol.

[39] Rathore S, Kumar B, Tehrani S, Khanra D, Duggal B, Chandra Pant D (2020). Cardiac rehabilitation: Appraisal of current evidence and utility of technology aided home-based cardiac rehabilitation. Indian Heart J.

[40] Read SA, Morton TA, Ryan MK (2015). Negotiating identity: a qualitative analysis of stigma and support seeking for individuals with cerebral palsy. Disabil Rehabil.

[41] Baykal Şahin H, Kalaycıoğlu E, Şahin M (2021). The effect of cardiac rehabilitation on kinesiophobia in patients with coronary artery Disease. Turk J Phys Med Rehabil.

[42] Williamson TM, Rouleau CR, Aggarwal SG, Arena R, Hauer T, Campbell TS (2021). The impact of patient education on knowledge, attitudes, and cardiac rehabilitation attendance among patients with coronary artery Disease. Patient Educ Couns.

[43] Neubeck L, Freedman SB, Clark AM, Briffa T, Bauman A, Redfern J (2012). Participating in cardiac rehabilitation: a systematic review and meta-synthesis of qualitative data. Eur J Prev Cardiol.

[44] Platz K, Kools S, Howie-Esquivel J (2023). Benefits, facilitators, and barriers of alternative models of Cardiac Rehabilitation: a qualitative systematic review. J Cardiopulm Rehabil Prev.

[45] Antoniou V, Pasias K, Loukidis N (2023). Translation, cross-cultural adaptation and psychometric validation of the Greek Version of the Cardiac Rehabilitation barriers Scale (CRBS-GR): what are the barriers in South-East Europe?. Int J Environ Res Public Health.

[46] Pedersen M, Overgaard D, Andersen I, Baastrup M, Egerod I (2017). Experience of exclusion: a framework analysis of socioeconomic factors affecting cardiac rehabilitation participation among patients with acute coronary syndrome. Eur J Cardiovasc Nurs.

[47] Okwose NC, O’Brien N, Charman S (2020). Overcoming barriers to engagement and adherence to a home-based physical activity intervention for patients with Heart Failure: a qualitative focus group study. BMJ Open.

[48] Yang Z, Liu S, Dai M, Zhang H (2021). Knowledge, attitude and practice of advance care planning among nursing interns: a mixed-methods approach. Nurse Educ Pract.

[49] Gulick V, Graves D, Ames S, Krishnamani PP (2021). Effect of a virtual reality-enhanced Exercise and Education intervention on Patient Engagement and Learning in Cardiac Rehabilitation: Randomized Controlled Trial. J Med Internet Res.

[50] Bäck M, Cider Ã, Herlitz J, Lundberg M, Jansson B (2016). Kinesiophobia mediates the influences on attendance at exercise-based cardiac rehabilitation in patients with coronary artery Disease. Physiother Theory Pract.

[51] Da Silva H, Pardaens S, Vanderheyden M, et al. Autonomic symptoms and associated factors in patients with chronic Heart Failure. Acta Cardiol. 2021;1–9. 10.1080/00015385.2021.2010953. 10.1080/00015385.2021.2010953 34886753

[52] Woodgate J, Brawley LR (2008). Self-efficacy for exercise in cardiac rehabilitation: review and recommendations. J Health Psychol.

[53] Keessen P, Kan KJ, Ter Riet G (2022). Impact of kinesiophobia on initiation of cardiac rehabilitation: a prospective cohort path analysis. BMJ Open.

[54] Fletcher JS, Banasik JL (2001). Exercise self-efficacy. Clin Excell Nurse Pract.

[55] Dubov A, Altice FL, Fraenkel L (2018). An information-motivation-behavioral skills model of PrEP uptake. AIDS Behav.

[56] Candelaria D, Kirkness A, Bruntsch C (2022). Exercise Self-efficacy improvements during Cardiac Rehabilitation: impact of Social disparities. J Cardiopulm Rehabil Prev.

[57] Goodyke MP, Hershberger PE, Bronas UG, Dunn SL (2022). Perceived Social Support and Heart Rate Variability: an integrative review. West J Nurs Res.

[58] Nielsen J, Duncan K, Pozehl B (2019). Patient-selected strategies for Post Cardiac Rehabilitation Exercise Adherence in Heart Failure. Rehabil Nurs.

[59] Ranaldi H, Deighan C, Taylor L (2018). Exploring patient-reported outcomes of home-based cardiac rehabilitation in relation to Scottish, UK and European guidelines: an audit using qualitative methods. BMJ Open.

[60] Heron N, Kee F, Donnelly M, Cardwell C, Tully MA, Cupples ME (2016). Behaviour change techniques in home-based cardiac rehabilitation: a systematic review. Br J Gen Pract.

